# Electrolyte Additive Strategies in Aqueous Zn‐Ion Batteries: Recent Advances and Prospects

**DOI:** 10.1002/advs.202522363

**Published:** 2026-03-20

**Authors:** Yuanze Yu, Hongmin Liu, Long Fan, Huakun Liu, Shixue Dou, Guoxiu Wang, Nana Wang, Zhongchao Bai

**Affiliations:** ^1^ Institute of Energy Materials Science (IEMS) University of Shanghai for Science and Technology Shanghai China; ^2^ Center for Instrument and Analysis University of Shanghai for Science and Technology Shanghai China; ^3^ Centre for Clean Energy Technology School of Mathematical and Physical Sciences Faculty of Science University of Technology Sydney Sydney NSW Australia

**Keywords:** aqueous zinc ion batteries, dendrites suppression, electrolyte additives, interfacial engineering

## Abstract

Aqueous zinc ion batteries (AZIBs) are considered one of the most promising energy storage systems due to their environmental friendliness, the intrinsic safety, and the abundance of Zn resources. However, using aqueous electrolytes presents many challenges for AZIBs, such as Zn dendrites, side reactions, and active material dissolution. Among the many solutions, electrolyte additives have emerged as a focal point in recent research due to their flexible structure, low cost, and strong regulating ability. Current discourse predominantly centers on the effect of additives on the electrode and electrode/electrolyte interface of AZIBs, while systematic evaluation and future perspectives on the types of additives remain underexplored. Therefore, it is necessary to systematically summarize electrolyte additive engineering and to further explore unresolved challenges. This review provides a comprehensive overview of electrolyte additives organized around three functional directions and presents the recent progress and representative strategies for anode protection additives, cathode stabilization additives, and electrolyte regulation additives. Finally, the paper summarizes the key issues in current research and looks at future directions. This paper aims to provide a theoretical foundation and practical guide for the research and application of high‐performance and long‐life AZIBs.

## Graphical abstract

This article provides a comprehensive overview of the current status and future development directions of AZIBs electrolyte additives in three aspects: stabilizing zinc anodes (uniform deposition, inhibition of dendritic crystals), protecting cathodes (structural stability, inhibition of dissolution), and enhancing electrolyte stability (wider electrochemical window, inhibition of side reactions). It highlights the potential of multifunctional and sustainable additives in achieving high‐performance, long‐life AZIBs.

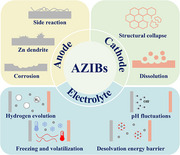

## 1 Introduction

The growing global demand for fossil energy is a major contributor to global warming and the greenhouse effect. With the aggravation of the energy crisis, reducing the accelerated consumption of fossil resources and developing clean, safe, and efficient large‐scale energy storage systems have become the key tasks of energy development in countries around the world [1, 2, 3, 4]. Conventional lithium‐ion batteries (LIBs) are essential in energy storage systems due to their high power and energy density [5, 6]. However, the shortage of Li resources and the use of flammable organic electrolyte systems have hindered the large‐scale application of LIBs [7, 8, 9]. Therefore, there is an urgent need to develop a new energy storage technology that is environmentally friendly, safe, and cost‐effective. Among the potential alternatives, aqueous zinc ion batteries (AZIBs) offer not only higher safety, but also their high theoretical capacity (820 and 5855 mAh cm^−3^) and low redox potential (−0.763 V vs. standard hydrogen electrode) [10, 11, 12]. In addition, the high abundance of Zn metal in the crust and the low processing cost make it naturally economically sustainable and suitable for the construction of a “low‐carbon battery system” [13, 14, 15].

**FIGURE 1:**
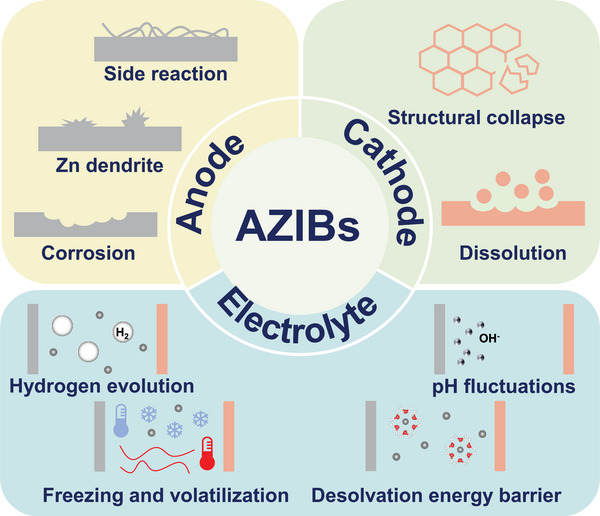
Unresolved issues exist in the anode, cathode, and electrolyte of AZIBs based on traditional aqueous electrolytes.

Although AZIBs exhibit significant application potential, there are a number of pressing issues that need to be addressed in the charging and discharging of AZIBs (Figure 1). The following types of problems are common at the electrolyte/electrode interface of AZIBs. (1) Uncontrolled growth of Zn dendrites. Sharp Zn dendrites can easily penetrate the separator, resulting in a short circuit of the cell [16]; (2) Side reactions such as the hydrogen evolution reaction (HER). The HER reduces the Coulombic Efficiency (CE) and is accompanied by changes in the pH of the electrolyte, triggering a buildup of by‐products [17]; (3) Dissolution and structural collapse of the cathode material. This can lead to the loss of active material and reduce the capacity of the cell [18]; (4) Destabilization of the electrolyte. The instability of the electrolyte leads to the accumulation of by‐products such as Zn_4_SO_4_(OH)_6_·xH_2_O, blocking the electrolyte/electrode interface, affecting ion transport and reaction kinetics [19]. The above problems not only limit the lifetime and reversibility of AZIBs, but also affect their reliability at extreme temperatures and high multiplicity conditions [20].

To address these challenges, researchers in various countries have conducted a variety of explorations, including separator design, Zn cathode surface design, and electrolyte optimization [21]. Functional separators are designed to address key electrochemical and structural issues that cannot be effectively dealt with by conventional separators [22, 23]. By introducing Zn‐friendly groups (e.g., carboxyl groups and amino groups) or functional coatings (e.g., graphene and MXene) on the surface of the separators, the Zn deposition behavior can be effectively regulated, thereby suppressing dendrite formation [24]. Alternatively, Zn^2^⁺ ions transport channels (e.g., metal–organic frameworks) can be constructed on the separators to realize the selective transport of Zn^2^⁺ ions and improve the CE [25]. However, the coating thickness is not easy to control, and the poor stability, as well as the difficulty and high cost of constructing the ion channels, seriously limit its large‐scale application [26, 27]. The Zn anode surface design is important for solving the problems of Zn dendrite growth, side reactions, and volume expansion that occur in Zn anodes during electrochemical cycling [28]. The construction of an artificial interfacial layer can inhibit HER well and provide good interfacial wettability [29, 30]. In addition, the design of a three‐dimensional (3D) structured Zn anode can effectively alleviate the problems caused by the current density concentration, small contact area, and volume change of a two‐dimensional (2D) planar Zn anode [31, 32]. Electrolyte optimization is a relatively simple and practical approach compared to complex separator design and Zn anode structure design [33]. Electrolyte optimization includes water‐in‐salt electrolytes, additive engineering, and gel/solid electrolytes [34]. Water‐in‐salt electrolytes (e.g., Zn(TFSI)_2_ ≥ 20 mol kg^−1^) are used to reduce the number of free H_2_O molecules by dramatically increasing the salt concentration so that Zn^2^⁺ ions are mainly coordinated to anions or a very small number of H_2_O molecules, which reduces the H_2_O activity and inhibits the HER [35]. However, this can lead to an increase in the viscosity and a deterioration of the ionic conductivity of the electrolyte. Gel electrolytes (e.g., PVA, PAM) are formed by wrapping the electrolyte with a polymer matrix to form a quasi‐solid structure to provide mechanical support, mitigate Zn electrode volume changes, and limit the exposure of Zn^2^⁺ ions to H_2_O [36, 37, 38]. Electrolyte additive engineering is the introduction of trace amounts of functional additives into conventional H_2_O‐based electrolytes [39]. The method is low cost, easy to operate, and adaptable, and is one of the frontiers of AZIBs research [40]. It is able to finely regulate the interfacial behavior and electrochemical reaction kinetics of Zn^2^⁺ ions without changing the main electrolyte system and cell structure [41]. The additives are rich in regulatory mechanisms, which can work through multiple mechanisms such as regulating Zn nucleation behavior, regulating the solvation structure of Zn^2^⁺ ions, and forming stable interfacial films [42]. This makes additive engineering a highly modular and tunable strategy for a wide range of anode/cathode systems and allows for the targeted design of molecular structures and functional groups for different problems [43].

There has been a great deal of research into electrolyte additives to optimize AZIBs. Specifically, Zhou et al. described the mechanism of the effect of different additives on AZIBs, while Zhi et al. reviewed the mechanisms by which various additives affect the structure of solvated sheaths [44, 45]. However, the existing reviews primarily focus on either specific electrode interfaces or solvation behavior without providing a comprehensive categorization of additive functions. In this review, we discuss electrolyte additives for different problems in AZIB, including protection of the anode, protection of the cathode, and stabilization of the electrolyte. The structural properties and functional mechanisms of different types of electrolyte additives are sorted out, and their effects on electrode interface behavior, electrolyte stability, and cell performance are summarized. Through these efforts, we expect to provide a scientific basis and research reference for the interface regulation, electrolyte innovative design, and industrialized application of AZIBs.

## 2 Additives for Anode Protection

### 2.1 Problems With Zn Anode and Formation Mechanisms

Zn metal anodes in AZIBs are prone to self‐corrosion behaviors, side reactions, and Zn dendrites growth during charging and discharging, which seriously affect the reversibility of Zn anodes. Self‐corrosion is the spontaneous chemical reaction of Zn metal with H⁺ ions in H_2_O or electrolyte in the absence of applied current, which is manifested by slow but continuous Zn dissolution and H_2_ gas release. The reaction process continues in the absence of current and is mainly driven by the anode potential of Zn. Its thermodynamics and kinetics are easier to realize, especially on Zn electrodes with more active sites or rough surfaces [46, 47]. OH^−^ ions from HER enrich at the local Zn anode surface, which will undergo further side reactions with anions such as Zn^2^⁺ and SO_4_^2^^−^ ions in the electrolyte to produce alkaline Zn sulfate (Zn_4_SO_4_(OH)_6_·xH_2_O) [48]. These by‐products are electrical insulators and are easily deposited on the surface of Zn to form a non‐conductive passivation layer, which in turn hinders the diffusion and electron transfer of Zn^2^⁺ ions, resulting in increased polarization of the electrode and decreased capacity utilization [49]. Both self‐corrosion and side reactions lead to disturbed Zn deposition behavior, inducing inhomogeneous deposition and Zn dendrites growth. Zn dendrites are dendritic, needle‐like, or hair‐like metallic microstructures formed by nonuniform deposition of Zn on the surface of the Zn anode during electrochemical cycling. The formation process is accompanied by localized deposition intensification, interface roughening, and “tip effect” [50, 51]. The formation of Zn dendrites is usually divided into three stages. Stage I is the initial nucleation stage. When an applied voltage drives the deposition of Zn^2^⁺ ions to the anode, Zn^2^⁺ ions first need to “nucleate” on the anode surface. Due to the presence of defects or impurities on the surface of the electrode, the electric field is not uniform, resulting in a concentration of nucleation sites [39]. Stage II is the nonuniform growth stage. The nucleated area will grow rapidly, and Zn^2^⁺ ions tend to migrate to the nucleated point, forming a positive feedback mechanism of “deposition first, strengthening later.” This is mainly driven by the “tip effect,” concentration polarization, and spatial limitation [52]. Finally, the dendrite aggregation and film penetration stage is reached. Multiple dendrites continue to grow in the vertical direction and eventually penetrate the separator, leading to separator rupture, cell short circuit, and even thermal runaway [53]. These problems not only limit the reversibility of AZIBs but also reduce their CE and cycle life.

### 2.2 Additive Strategies and Representative Compounds

The introduction of electrolyte additives in AZIBs can effectively regulate the deposition behavior of Zn^2^⁺ ions and enhance the stability and kinetics of their solvation structures [54]. This is primarily manifested in: (1) Molecules with adsorption activity (e.g., those containing ─COOH/─OH/─SO_3_^−^ groups) can preferentially anchor on the Zn surface, uniformly distributing interfacial charges to suppress the “tip effect” and promote uniform nucleation [55]; (2) Certain additives (e.g., those containing ─C═O/─COO^−^ group, nitrogen‐containing groups) partially replace H_2_O molecules in the Zn^2^⁺ ion solvation shell or reorganize hydrogen bonding (H‐bonding) networks, thereby lowering desolvation energy barriers and reducing H_2_O activity, synergistically suppressing side reactions like HER [56]; (3) Film‐forming additives (e.g., those containing ─OH/─O─/─NH_2_ groups) can in situ induce the formation of an ion‐conductive anode/electrolyte interface (AEI) layer. This layer prevents direct contact between the Zn electrode and electrolyte, buffers local pH, and inhibits the accumulation of byproducts such as basic Zn salts [57]. Through these synergistic effects, additives suppress self‐corrosion, side reactions, and Zn dendrite growth while simultaneously reducing over‐potentials and enhancing CE.

**FIGURE 2:**
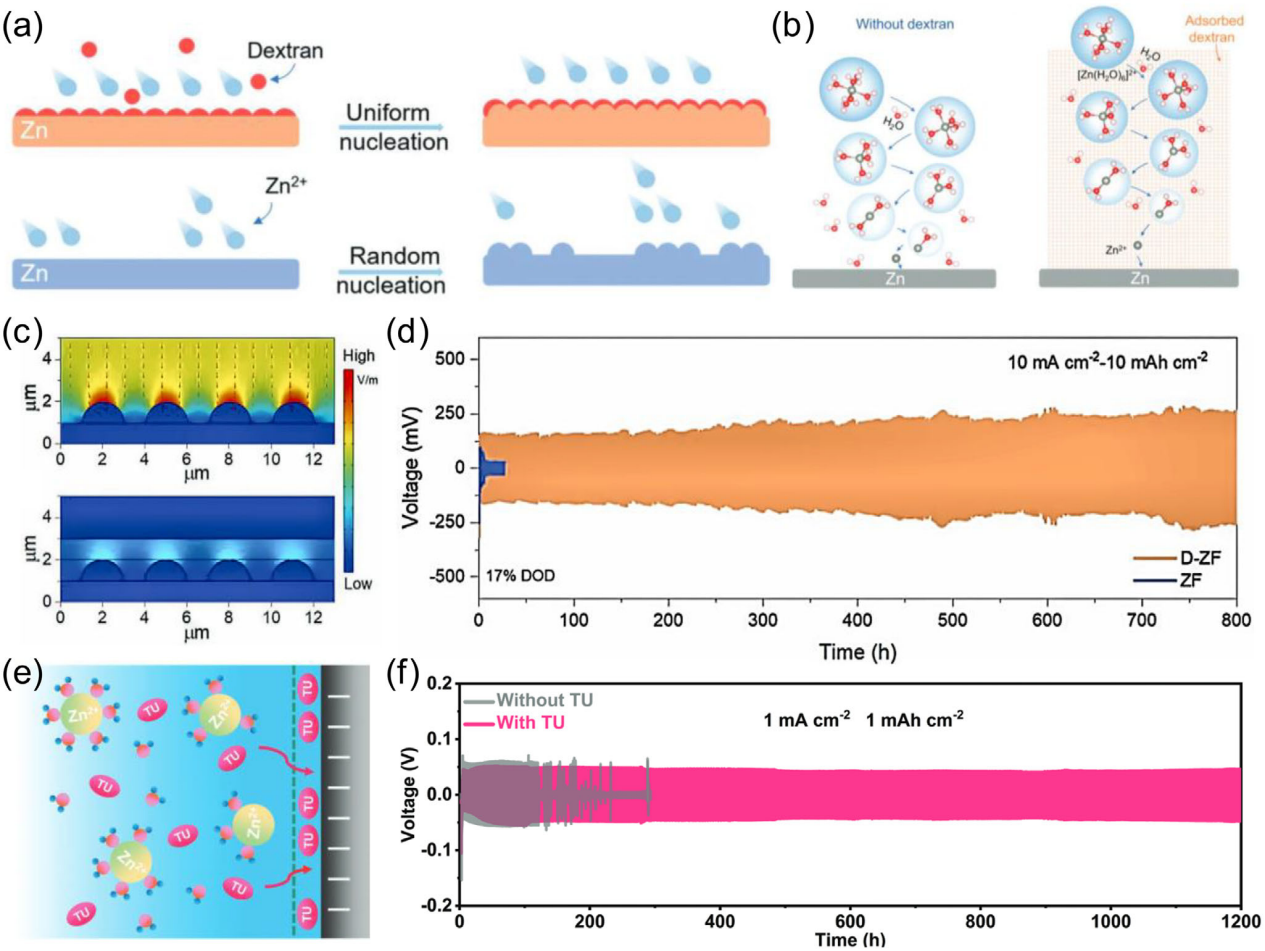
(a) Schematic illustrations of Zn plating behaviors in different electrolytes. Reprinted with permission from Ref [58]. Copyright 2023 Wiley‐VCH. (b) Schematic illustrations of the desolvation processes in different electrolytes. Reprinted with permission from Ref [58]. Copyright 2023 Wiley‐VCH. (c) Simulated electric field distributions at the Zn electrode/electrolyte interface in different electrolytes. Reprinted with permission from Ref [58]. Copyright 2023 Wiley‐VCH. (d) Time–voltage profiles of the Zn||Zn symmetric cells. Reprinted with permission from Ref [58]. Copyright 2023 Wiley‐VCH. (e) Schematic illustration of ZnSO_4_ electrolyte after introducing TU. Reprinted with permission from Ref [59]. Copyright 2022 Wiley‐VCH. (f) Cycle performances of the Zn||Zn symmetric cells. Reprinted with permission from Ref [59]. Copyright 2022 Wiley‐VCH.

Specifically, Li et al. incorporated a dextran additive that combines four functions into AZIBs electrolyte [58]. First, dextran in situ induces the formation of an ion‐conductive and mechanically compliant AEI layer, preventing direct contact between the Zn anode and electrolyte while buffering local pH fluctuations and suppressing by‐product generation (Figure 2a). Second, dextran addition lowers the desolvation energy barrier for Zn^2^⁺ ions, facilitating smoother desolvation‐electron transfer‐surface diffusion and enhancing Zn^2^⁺ ions transport and deposition efficiency (Figure 2b). Third, the abundant ─OH/ether bond sites in dextran enable multi‐point adsorption on the Zn metal surface, homogenizing interfacial charge distribution and weakening localized electric field concentration. This suppresses the “tip effect” at its source, guiding uniform in‐plane nucleation and dense lateral growth (Figure 2c). Finally, dextran induces preferential Zn growth along the (0002) crystal plane, helping to inhibit dendrites formation. Consequently, the Zn||Zn symmetric cell achieved a long cycling life of 800 h at a current density of 10 mA cm^−2^ in the dextran environment, with a cumulative plating capacity reaching 4000 mAh cm^−2^ (Figure 2d).

**FIGURE 3:**
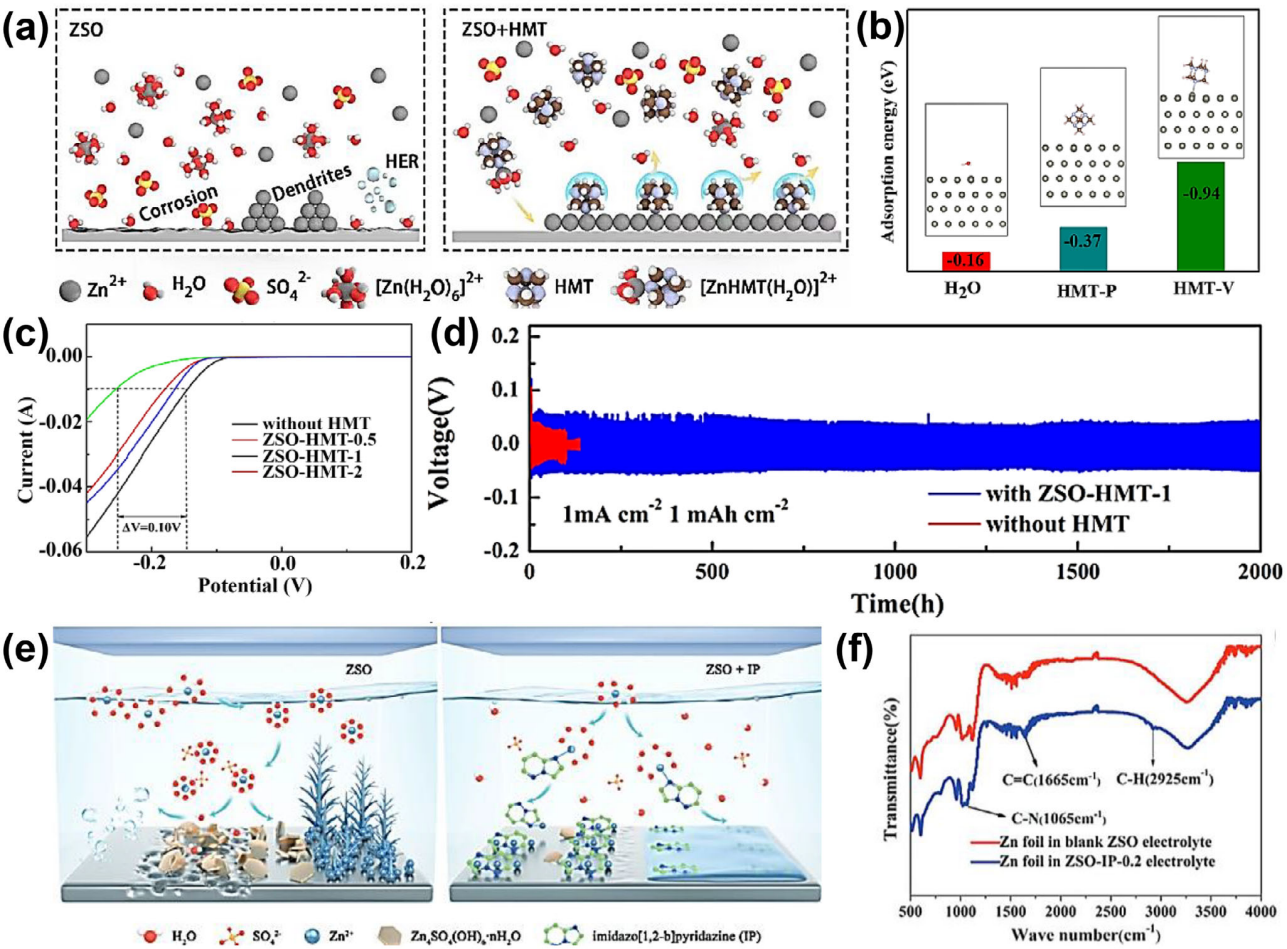
(a) Protective mechanisms of the HMT additive on Zn anodes. Reprinted with permission from Ref [60]. Copyright 2023 Elsevier. (b) Adsorption energies of H_2_O and HMT molecules on Zn (002) crystal plane. Reprinted with permission from Ref [60]. Copyright 2023 Elsevier. (c) Linear sweep voltammetry (LSV) curves of different electrolytes. Reprinted with permission from Ref [60]. Copyright 2023 Elsevier. (d) Time–voltage profiles of Zn||Zn symmetric cells. Reprinted with permission from Ref [60]. Copyright 2023 Elsevier. (e) The protection mechanisms of Zn anodes in ZnSO_4_ with/without IP additive. Reprinted with permission from Ref [61]. Copyright 2024 Wiley‐VCH. (f) FTIR spectra of Zn foils in the electrolyte with/without IP. Reprinted with permission from Ref [61]. Copyright 2024 Wiley‐VCH.

Qin et al. introduced thiourea (TU) into ZnSO_4_ to construct a metal–molecular interface for stabilizing Zn anodes [59]. TU replaces interfacial H_2_O through Zn–S chemisorption, forming a low‐H_2_O‐activity interface that suppressed corrosion and HER; Its N/S lone pair electron sites coordinate Zn^2^⁺ ions, restructuring the dissolution sheath of Zn^2^⁺ ions (Figure 2e); Simultaneously, it flattens the electric field, increases uniform nucleation sites, and lowers the nucleation barrier, inducing dense, dendrite‐free deposition while suppressing the accumulation of the byproducts. As a result, the Zn||Zn symmetric cell achieves a 1200 h cycle life at 1 mA cm^−^^2^ (Figure 2f). Gu et al. used Hexamethylenetetramine (HMT) as an electrolyte additive to in situ construct a Zn‐HMT composite film protective layer on the surface of the Zn anode (Figure 3a) [60]. Theoretical calculations show that HMT tends to adsorb more vertically on the Zn surface, and electrons are transferred from the ─NR group in HMT to the Zn surface to build stable Zn─N bonds (Figure 3b). This stronger chemical adsorption modulates the ion flux, inducing uniform lateral growth of Zn. In addition, the protective layer containing hydrophobic alkyl tails prevents the coordination of H_2_O with Zn^2+^ ions, which affects the solvation structure of Zn^2+^ ions and significantly inhibits the HER (Figure 3c). As a result, the Zn||Zn symmetric cell with HMT electrolyte (1 mg mL^−1^) achieves a cycle stability of more than 2000 h (Figure 3d).

**FIGURE 4:**
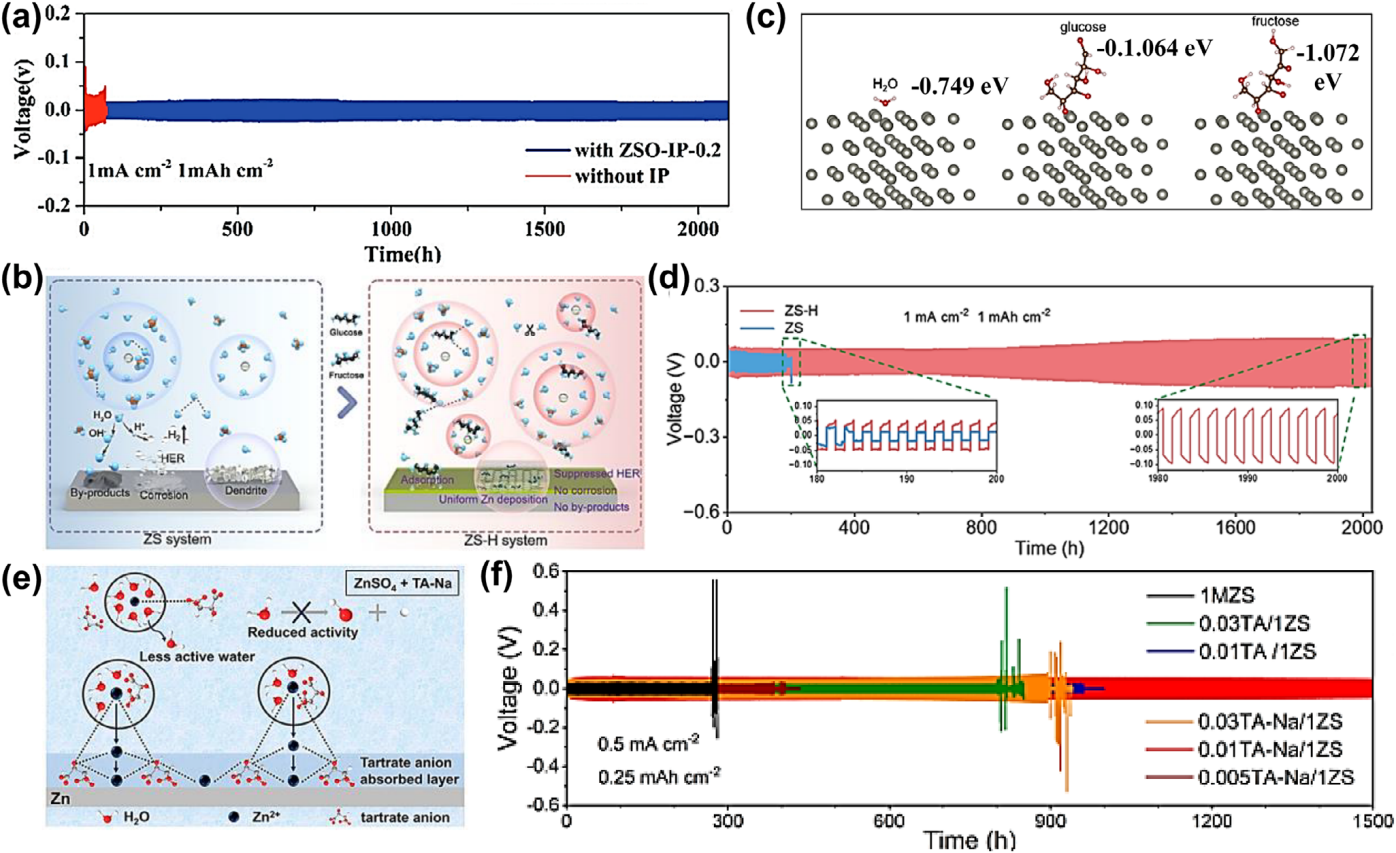
(a) Time–voltage profiles of the Zn||Zn symmetric cells. Reprinted with permission from Ref [61]. Copyright 2024 Wiley‐VCH. (b) Schematic illustrations of Zn^2+^ ion solvation structures and interfacial chemistry at Zn anodes. Reprinted with permission from Ref [62]. Copyright 2024 Elsevier. (c) Adsorption energies of H_2_O, glucose, and fructose molecules on the Zn anode. Reprinted with permission from Ref [62]. Copyright 2024 Elsevier. (d) Long‐term cycle stability of Zn||Zn symmetric cells. Reprinted with permission from Ref [62]. Copyright 2024 Elsevier. (e) Schematic illustrations of the Zn^2+^ ion solvation structure and the interfacial reaction between the Zn anode and the electrolyte. Reprinted with permission from Ref [34]. Copyright 2023 American Chemical Society. (f) Time–voltage profiles of the Zn||Zn symmetric cells with different electrolytes. Reprinted with permission from Ref [34]. Copyright 2023 American Chemical Society.

Zhang et al. added imidazo[1,2‐b]pyridazine (IP) to 2 m ZnSO_4_ electrolyte to stabilize the Zn anode through a dual shielding–anchoring effect [61]. On one hand, in the slightly acidic ZnSO_4_ solution, IP with multiple N‐containing functional groups can adsorb on the surface of the Zn anode and a H_2_O molecule in the Zn^2+^ ion solvation structure can be replaced by an IP molecule to form [Zn(H_2_O)_5_(IP)]^2+^ (Figure 3e). This reduces the H_2_O molecules in the solvation structure of Zn^2+^ ions and lowers the activity of H_2_O molecules, thereby inhibiting the occurrence of side reactions such as HER. On the other hand, FTIR analysis was performed on Zn anodes plated/stripped in IP electrolyte. The appearance of new peaks of C─N bond and the stretching vibration of C═C and C─N bonds can be observed (Figure 3f), which indicates that the ionized IP can adsorb on the surface of the Zn anode, modulate the Zn^2+^ ion flow to guide the homogeneous deposition of Zn^2+^ ions, and inhibit the growth of Zn dendrites. Consequently, the Zn||Zn symmetric cell can cycle stably for up to 2200 h at an optimal IP concentration of 0.2 mg mL^−1^ (Figure 4a).

Wang et al. used natural honey as a low‐cost, nontoxic electrolyte additive (Figure 4b) [62]. As shown in Figure 4c, the adsorption energies of glucose and fructose on the Zn metal are all higher than those of water molecules. This indicates that an adsorption layer can form on the Zn electrode surface, thereby slowing down dendrite growth. Simultaneously, the glucose and fructose in honey are rich in hydroxyl groups, which can form a more stable coordination structure with Zn^2^⁺ ions, attenuate the interaction between Zn^2^⁺‐H_2_O and Zn^2^⁺‐SO_4_^2^^−^, and promote the Zn^2^⁺ ion desolvation. The addition of honey significantly enhances the corrosion resistance of the Zn electrode and promotes uniform deposition, and the lifetime of the Zn||Zn symmetric cell is increased from 160 to 2000 h (Figure 4d). Liang et al. proposed sodium tartrate (TA‐Na) as a bifunctional additive, simultaneously regulating the anode/electrolyte interface and the Zn^2^⁺ ion dissolution shell [34]. The tartrate anion preferentially adsorbs onto Zn surfaces and guides uniform nucleation; its nucleophilic carboxylate coordinates with Zn^2^⁺ ions, partially displacing H_2_O molecules from [Zn(H_2_O)_6_]^2^⁺, thereby reducing H_2_O activity and desolvation energy barriers while inhibiting HER (Figure 4e). This strategy enabled AZIBs to achieve stable cycling exceeding 1500 h (Figure 4h).

### 2.3 Current Challenges and Future Outlooks

Although electrolyte additives have shown significant benefits in regulating the Zn anode interface in batteries, they still face many challenges in practical applications. First, there is a lack of systematic and accurate theoretical explanation of the specific mechanism of additives in the regulation of Zn^2+^ ion solvation structure and nucleation induction, and the limitations of in situ characterization and simulation further restrict the in‐depth understanding of the mechanism [43]. Second, most of the additive‐induced protective layer structures are loose and difficult to control the thickness, which cannot maintain the stability under long cycling or high multiplicity conditions [63]. In addition, it is difficult to control the concentration of additives, which are easily consumed during the cycling process, leading to the decay of the protective effect [64]. Some additives may even introduce new side reactions, affecting the synergistic stability of the whole battery system, limiting their popularization and application in actual batteries [65].

In the future, the design and application of electrolyte additives for anode protection can focus on the following aspects: first, developing compounds with multifunctional synergistic regulation ability, such as composite additives with Zn affinity and hydrophobicity at the same time [66]; second, constructing stable interfacial membranes that can be self‐assembled or have self‐repairing ability, and improving the long‐term integrity and selectivity of the interface [67]; third, enhancing the integration of in situ characterization with the calculation of the first principles, and deepening the microscopic understanding of additive behavior and interfacial mechanisms [68]; fourth, to establish a full cell evaluation system that is closer to the actual working conditions, and to promote the engineering application of high‐efficiency and sustainable additives [69].

## 3 Additives for Cathode Protection

### 3.1 Challenges of Cathode Dissolution and Structural Instability

In AZIBs, the cathode is the key material to carry reversible intercalation/decalcification of Zn^2^⁺ ions, and its performance directly affects the energy density, multiplicity performance, and cycle life of the cell. Ideal cathode materials need to be characterized by high specific capacity, good reversible embedding ability, water stability, and structural stability [70, 71]. Common cathode materials include Mn‐based materials, V‐based materials, and Prussian blue analogs (PBAs) [72]. Mn‐based materials (e.g., MnO_2_, Mn_3_O_4_, MnOOH) store Zn mainly through Zn^2^⁺ or H⁺ ions intercalation and de‐embedding, accompanied by Mn valence state changes (Mn⁴⁺ ↔ Mn^3^⁺ ion) [73, 74, 75]. V‐based materials (e.g., V_2_O_5_·nH_2_O, VO_2_) store Zn by lamellar structure insertion of Zn^2^⁺ or H⁺ ions accompanied by V⁵⁺/V⁴⁺ ions reduction/oxidation [76, 77]. The PBAs (e.g., Na_x_NiFe(CN)_6_, ZnHCF) are an open 3D framework structure, which facilitates rapid Zn^2^⁺ ions migration [78, 79].

Despite the evolutionary optimization of cathode materials, there are still some issues that constrain their performance. Structural instability: most cathode materials are layered or tunneled, relying on intracrystalline space to accommodate Zn^2^⁺ ions (e.g., MnO_2_, V_2_O_5_·nH_2_O). Whereas Zn^2^⁺ ions have a large radius (∼0.74 Å) and usually exist as [Zn(H_2_O)_6_]^2^⁺, intercalation is accompanied by volume expansion and lattice stress accumulation. Repeated Zn^2^⁺ ions intercalation/decalcification during cycling leads to irregular changes in the layer spacing, and irreversible structural collapse or crystalline phase transition occurs after long cycles [80, 81, 82]. Cathode material dissolution: Some cathode materials (e.g., MnO_2_, V_2_O_5_) have a dissolution–recombination equilibrium in acidic or neutral environments. During cycling, Mn/V elements are detached from the crystals to form Mn^2^⁺/VO_2_⁺ ions and enter the electrolyte, resulting in loss of active material [83, 84, 85]. Both collapse and dissolution of the cathode material structure lead to loss of active sites and reduction of the reactive region, ultimately leading to capacity decay [86].

### 3.2 Additive‐Driven Interfacial Engineering

Electrolyte additives also play a crucial role in enhancing the performance of AZIB cathodes. Certain additives can enhance the solubility of specific elements in cathode materials by intervening in their bulk chemical environment, establishing a “dissolution–redeposition” dynamic equilibrium. Alternatively, they may act as lattice “pillars” or participate in lattice rearrangement to enhance crystal stability, thereby mitigating irreversible dissolution and preventing capacity decay [84]. Additionally, film‐forming molecules can preferentially undergo electrochemical decomposition on the cathode surface during the battery's initial charging cycle, forming an ion‐conductive and hydrophobic cathode/electrolyte interface (CEI) layer in situ. This layer restricts solvent co‐intercalation and side reactions while inhibiting the entry and exit of H_2_O from the lattice, thereby stabilizing the solid–liquid interface, mitigating pH fluctuations, and preventing structural collapse of the cathode material [89].

**FIGURE 5:**
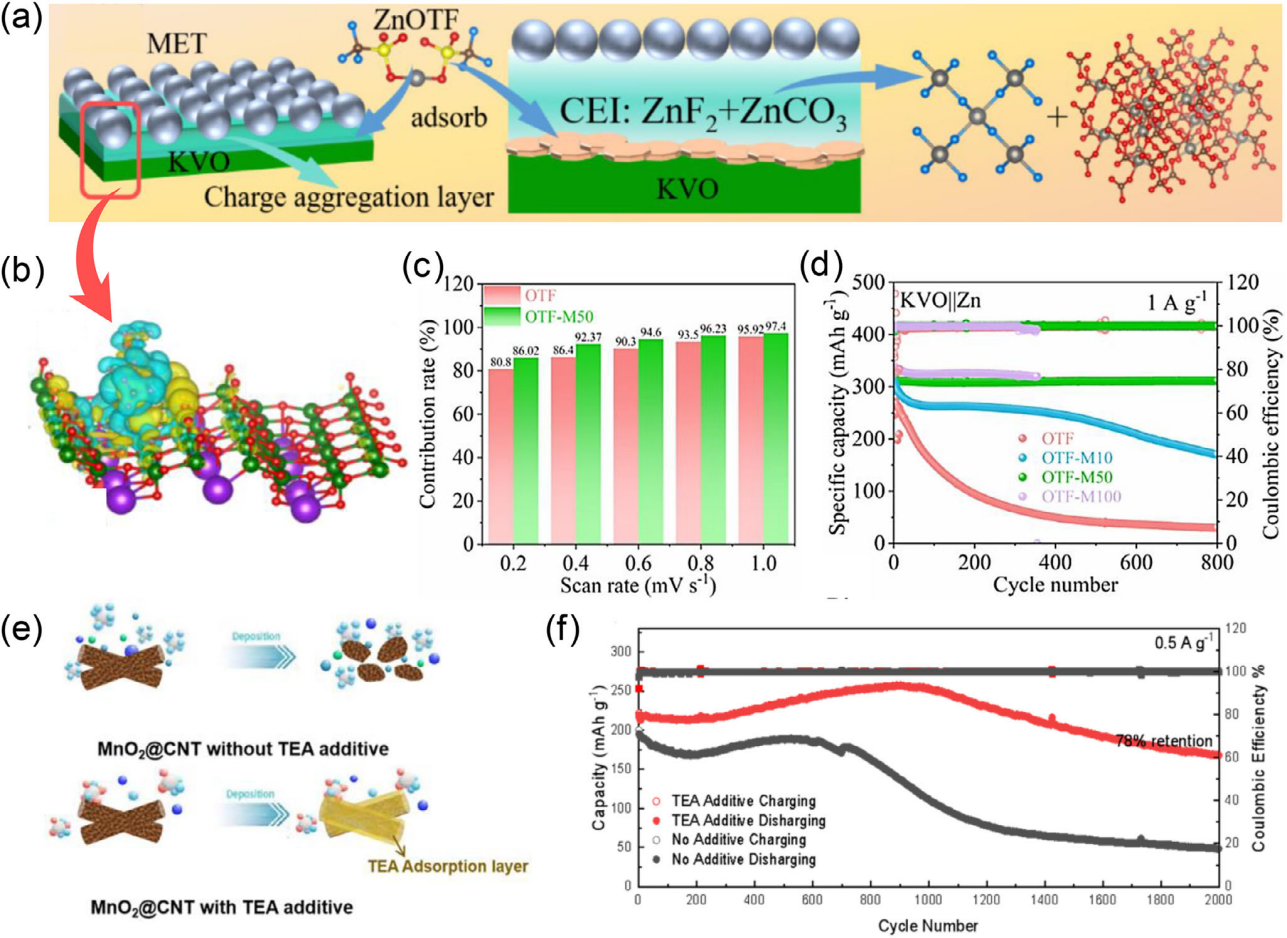
(a) The schematic diagrams of the KVO surface electronic state and CEI formation process. Reprinted with permission from Ref [87]. Copyright 2024 Elsevier. (b) Electron density difference diagram of KVO‐MET. Reprinted with permission from Ref [87]. Copyright 2024 Elsevier. (c) Contribution of pseudo capacitance at different scan rates. Reprinted with permission from Ref [87]. Copyright 2024 Elsevier. (d) Cycling performances of KVO||Zn full cells. Reprinted with permission from Ref [87]. Copyright 2024 Elsevier. (e) Schematic illustrations of the mechanisms of MnO_2_@CNT with/without TEA. Reprinted with permission from Ref [88]. Copyright 2022 Royal Society of Chemistry. (f) Cycling performances at a current density of 0.5 A g^−1^. Reprinted with permission from Ref [88]. Copyright 2022 Royal Society of Chemistry.

Zhang et al. introduced a bifunctional electrolyte additive, methionine (MET), aiming to solve the problem of the collapse and dissolution of V‐based cathode structure [87]. The protection of MET for the cathode is mainly reflected in the following aspects: First, it adsorbs onto the cathode surface to induce the in situ formation of a ZnF_2_‐enriched CEI, thereby preventing H_2_O in the electrolyte from corroding the cathode (Figure 5a). Second, it increases the dissolution energies of K and V elements while reducing the risk of structural dissolution. Finally, it enhances the capture capacity for electron states and Zn^2^⁺ ions, increases charge transfer rates, and boosts the proportion of pseudo capacitance contribution (Figure 5b,c), thereby maintaining framework stability. Consequently, the K_2_V_8_O_21_ (KVO)||Zn full cell exhibits a high capacity retention rate of 99.8% after 800 cycles (Figure 5d). Lin et al. used triethanolamine (TEA) as an electrolyte additive [88]. TEA can adsorb on the surface of MnO_2_@CNT electrodes to form CEI, preventing Mn^2^⁺ ion dissolution while enhancing Zn^2^⁺ ion diffusion at the cathode interface (Figure 5e). As shown in Figure 5f, the Zn||MnO_2_@CNT full cell can operate stably for 2000 cycles with a capacity retention up to 78%.

**FIGURE 6:**
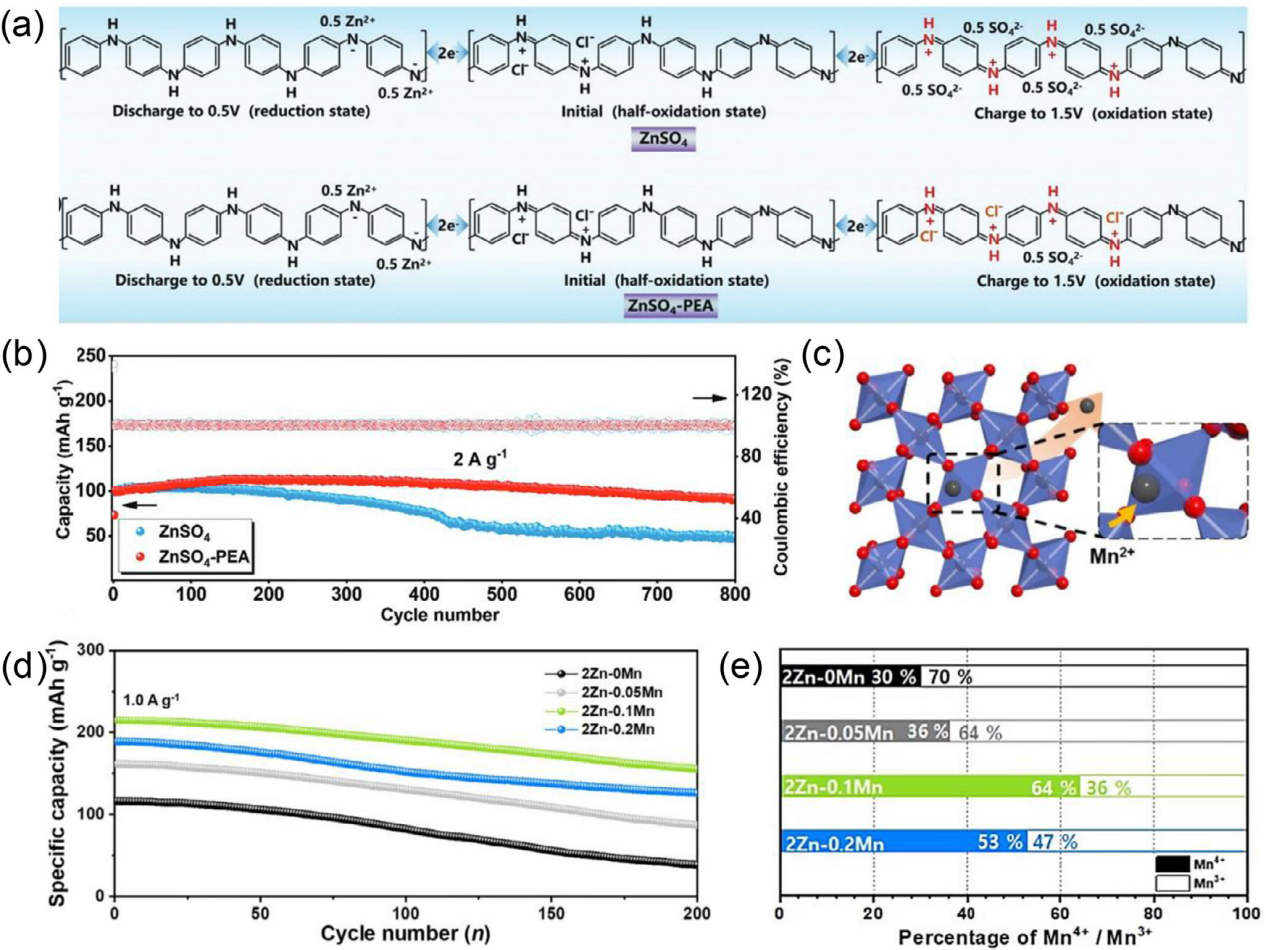
(a) Redox processes of PANI in different electrolytes. Reprinted with permission from Ref [90]. Copyright 2023 Wiley‐VCH. (b) Cycling performances of Zn||PANI full cells. Reprinted with permission from Ref [90]. Copyright 2023 Wiley‐VCH. (c) Schematic of the excellent structural stability of the MnO_2_ cathode after cycling. Reprinted with permission from Ref [91]. Copyright 2022 John Wiley & Sons Ltd. (d) Cycling performances of Zn||MnO_2_ full cells at a current density of 1.0 A g^−1^. Reprinted with permission from Ref [91]. Copyright 2022 John Wiley & Sons Ltd. (e) The percentage of Mn^4+^/Mn^3+^ ions in MnO_2_ cathodes. Reprinted with permission from Ref [91]. Copyright 2022 John Wiley & Sons Ltd.

Wang et al. discovered that 1‐phenylethylamine hydrochloride (PEA) can protect organic polyaniline (PANI) cathodes through anion regulation [90]. Specifically, during charging, Cl^−^ ions supplied by PEA replace SO_4_^2^^−^ ions inserted into oxidized PANI chains (Figure 6a). Due to the lower average hydration coordination number of Cl^−^ ions (6.7 vs. 12.5 for SO_4_^2^^−^ ions), H_2_O molecules surrounding the oxidized PANI are reduced. This suppresses H_2_O‐induced side reactions and enhances the electrochemical stability of PANI. Therefore, the specific capacity of the Zn||PANI full cell remains 91.8 mAh g^−1^ after 800 cycles of long cycling (Figure 6b). An et al. proposed the introduction of MnSO_4_ additives into the electrolyte to protect the β‐MnO_2_ cathode [91]. The externally added Mn^2^⁺ ions chemically inhibit the dissolution of active manganese through a “self‐healing” mechanism, mitigating Jahn–Teller‐induced structural degradation. This preserves the 1 × 1 channel framework and stable ion transport (Figure 6c). The Mn^2^⁺ ion additive also suppresses the conversion of Mn⁴⁺ ions to Mn^3^⁺ ions, preventing the formation of inert byproducts such as ZnMn_2_O_4_ and preserving electrode activity (Figure 6e). This significantly enhances the battery's cycling stability, achieving approximately 72% capacity retention after 200 cycles at 1.0 A g^−1^ (Figure 6d).

**FIGURE 7:**
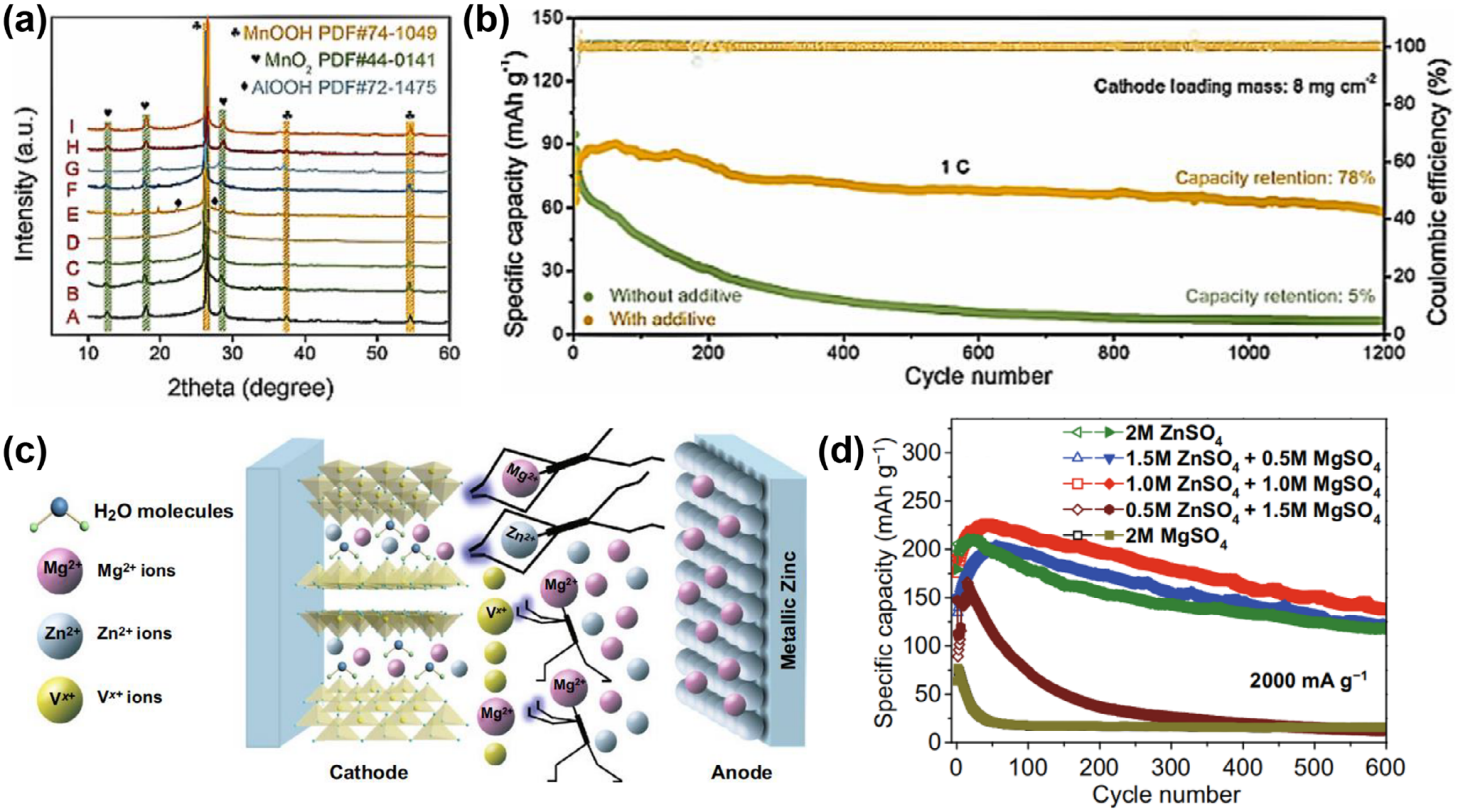
(a) XRD patterns of the MnO_2_ cathode in Al_2_(SO_4_)_3_ electrolyte. Reprinted with permission from Ref [92]. Copyright 2025 Owner Societies. (b) Long‐term cycling stability of Zn||MnO_2_ full cells with/without Al_2_(SO_4_)_3_. Reprinted with permission from Ref [92]. Copyright 2025 Owner Societies. (c) Scheme of the Mg^2+^ ion functional mechanism. Reprinted with permission from Ref [93]. Copyright 2020 Springer Nature. (d) Cycling performances of Zn||MgVO full cells at current density of 2000 mA g^−1^ in different electrolytes. Reprinted with permission from Ref [93]. Copyright 2020 Springer Nature.

Yang et al. introduced Al_2_(SO_4_)_3_ as an additive in ZnSO_4_ electrolyte, protecting the MnO_2_ cathode through solvation and interfacial synergistic effects [92]. Strongly hydrated Al^3^⁺ ions can “absorb H_2_O” and modulate local proton activity, inhibiting Mn^3^⁺ ion disproportionation. Simultaneously, XRD revealed that Al^3^⁺ ions reversibly participate in the MnO_2_ lattice structure, regulating crystal phase transitions through the AlOOH formation/transformation. This stabilizes the MnO_2_/MnOOH framework and reduces irreversible Mn dissolution (Figure 7a). Consequently, the MnO_2_ cathode achieved a capacity retention rate of 78% at a high loading of 8.0 mg cm^−^^2^ (Figure 7b). Li et al. protected the Mg_x_V_2_O_5_·nH_2_O (MgVO) cathode by introducing MgSO_4_ additives into ZnSO_4_ [93]. Dissolved Mg^2^⁺ ions establish a dissolution–recombination equilibrium at the electrolyte/cathode interface, inhibiting the continuous dissolution of vanadate and achieving a “self‐healing” interface. Simultaneously, Mg^2^⁺ ions act as an interlayer “pillar,” maintaining the V─O framework during cycling and facilitating rapid Zn^2^⁺ ions diffusion (Figure 7c). The synergistic intercalation/deintercalation of Zn^2^⁺/Mg^2^⁺ ions reduces polarization and optimizes kinetics. Consequently, at a current density of 2 A g^−1^, the Zn||MgVO full cell exhibits an 84% capacity retention rate after 600 long‐cycle tests (Figure 7d).

### 3.3 Current Challenges and Future Outlooks

Although electrolyte additives have shown significant potential to enhance the electrochemical stability of AZIBs cathode materials, they still face many challenges in practical applications [94]. First, there are significant fitness differences between different additives and specific cathode materials due to the essential differences in the dissolution and redox mechanisms of various cathode materials [95]. Therefore, additives should be designed specifically for different cathode materials. For example, Mn‐based materials face issues of Mn^3^⁺ ions dissolution and phase transitions, so additive design should focus on “self‐healing” and Mn^2^⁺ ions compensation [91]. V‐based materials encounter problems with layered structure collapse and V dissolution, potentially requiring “support” ions and interfacial passivation [87]. For PBAs, preventing side reactions caused by lattice H_2_O requires desiccants and lattice stabilizers. Second, many additives may be deactivated by consumption, electrode adsorption, or interfacial side reactions during the charging and discharging process, resulting in a gradual weakening of the protective effect [96]. In addition, although some additives can effectively improve the performance of the cathode, but at the same time may cause adverse effects on the Zn anode, such as triggering HER or changing the deposition morphology of Zn, which ultimately reduces the cycle life of the cell [97].

In the future, electrolyte additives still have a broad research space in cathode protection. First, there is an urgent need to develop functional additives with material specificity, which can precisely regulate the dissolution mechanism of specific cathode materials [98]. Second, it is necessary to induce in situ or non‐in situ generation of stable and conductive interfacial films (e.g., hydration film or inorganic passivation layer) to effectively isolate the cathode from the electrolyte and improve the interfacial stability [99]. Finally, theoretical calculations (e.g., density functional theory (DFT) calculation), MD simulation, and in situ characterization techniques (e.g., in situ XRD, Raman spectroscopy) are also of key significance for revealing the additive's mechanism of action and guiding molecular design [100].

## 4 Additives for Electrolyte Stability

### 4.1 Instability of Aqueous Electrolytes

Aqueous electrolyte is an ionic conductor system composed of H_2_O molecules as a solvent and soluble Zn salt (e.g., ZnSO_4_, Zn(CF_3_SO_3_)_2_, ZnCl_2_) added as a transmission medium for Zn^2^⁺ ions in cells, which has the advantages of high safety, low cost, and high ionic conductivity [101, 102]. However, it still faces a number of problems in long‐term operation: (1) Narrow electrochemical stabilization window. During the charging process, Zn^2^⁺ ion is reduced to Zn on the electrode surface, but at the same time, due to the high electrochemical activity of the H_2_O molecules, when the cell is higher than the theoretical decomposition voltage of H_2_O, the HER and oxygen evolution reaction (OER) are prone to occur, which leads to the expansion of the cell or even an explosion [103]. (2) Desolvation energy barrier. Zn^2^⁺ ions will form a stable [Zn(H_2_O)_6_]^2^⁺ coordination structure in H_2_O, and the desolvation process will result in a slow migration rate of Zn^2^⁺ ions and the insertion kinetic hysteresis, and requires a high energy barrier [104]. (3) Dramatic local pH fluctuations. Zn^2^⁺ ion releases two OH^−^ ions during reduction to Zn, and the HER continues to consume H⁺ ions, leading to increased local alkalinity and inducing Zn_4_SO_4_(OH)_6_·xH_2_O, Zn(OH)_2_, and other by‐products deposition [105]. (4) Poor adaptability to a wide temperature domain. H_2_O as a solvent is easy to freeze at low temperatures and volatile at high temperatures, limiting the application of cells in cold or high temperature environments [106].

### 4.2 Representative Electrolyte‐Stabilizing Additives

**FIGURE 8:**
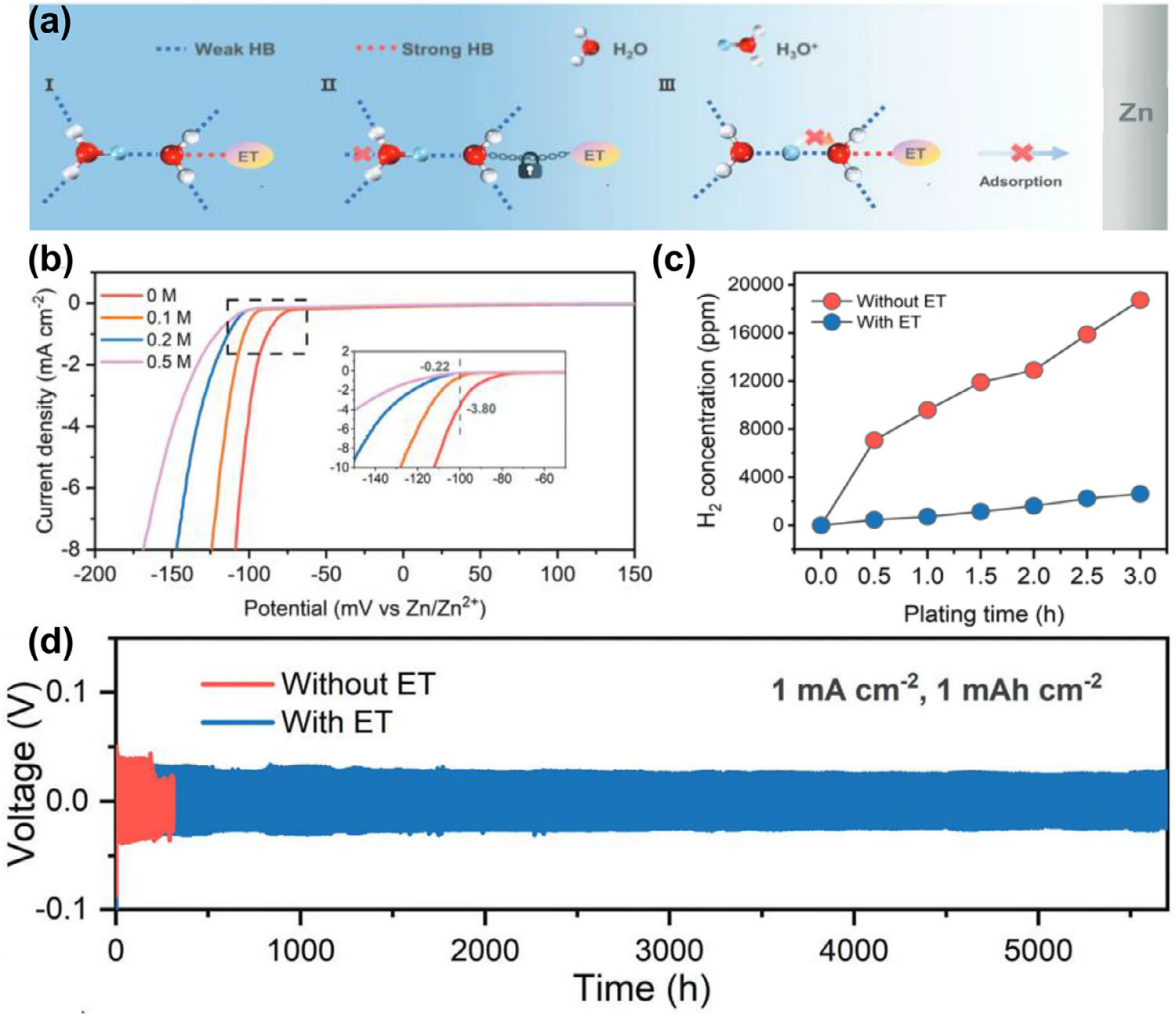
(a) Schematic illustration of the proton transfer through H‐bonds in the electrolyte with ET. Reprinted with permission from Ref [107]. Copyright 2024 Wiley‐VCH. (b) LSV curves of Zn||Ti cells. Reprinted with permission from Ref [107]. Copyright 2024 Wiley‐VCH. (c) The evolution of H_2_ concentration. Reprinted with permission from Ref [107]. Copyright 2024 Wiley‐VCH. (d) Time–voltage profiles of the Zn||Zn symmetric cells with/without ET. Reprinted with permission from Ref [107]. Copyright 2024 Wiley‐VCH.

In recent years, researchers have proposed various functional additives. The core contradiction in aqueous electrolytes lies in highly reactive free H_2_O molecules. Additive molecules can insert themselves into and disrupt the originally continuous H‐bonding network between H_2_O molecules, forming stronger or more complex H‐bonds with them [107]. This kinetically inhibits the HER. Simultaneously, it significantly reduces the overall activity and reactivity of free H_2_O molecules. Furthermore, additives containing strong coordinating atoms coordinate with Zn^2^⁺ ions, reducing the number of active H_2_O molecules directly coordinating with Zn^2^⁺ ions. Certain low‐temperature additives can also disrupt water's “ordering,” lowering the electrolyte's freezing point to remain liquid at temperatures below freezing [110]. High‐temperature additives, conversely, firmly “lock” H_2_O molecules through stronger coordination or intermolecular forces, enhancing the electrolyte's thermal stability [111]. For example, Tang et al. introduced ectoine (ET) with “kosmotrope” (Greek meaning order) effect into AZIBs [107]. When proton transfer occurs, a H‐bond in the H_2_O molecule that receives the proton breaks, and the O atom in the H_3_O^+^ ion that provides the proton forms a new H‐bond, which allows the proton to jump between the two H_2_O molecules (Figure 8a). However, after the addition of ET, the ET molecule has highly polar active sites, which can form stronger H‐bonds with H_2_O molecules, thus enhancing the energy barrier for H‐bond reconstruction and hindering the proton jumps between H_2_O molecules. Thus, effectively inhibiting the occurrence of HER (Figure 8b). H_2_ production during galvanization was detected using in situ electrochemical gas chromatography (EC‐GC). As shown in Figure 8c, in the electrolyte without ET, the total amount of H_2_ precipitates from the Zn anode is more than seven times that of the ET‐containing electrolyte after 3 h of plating. The inhibitory effect of ET on HER is further demonstrated. And Zn||Zn symmetric cell lasts more than 27 times longer (Figure 8d).

**FIGURE 9:**
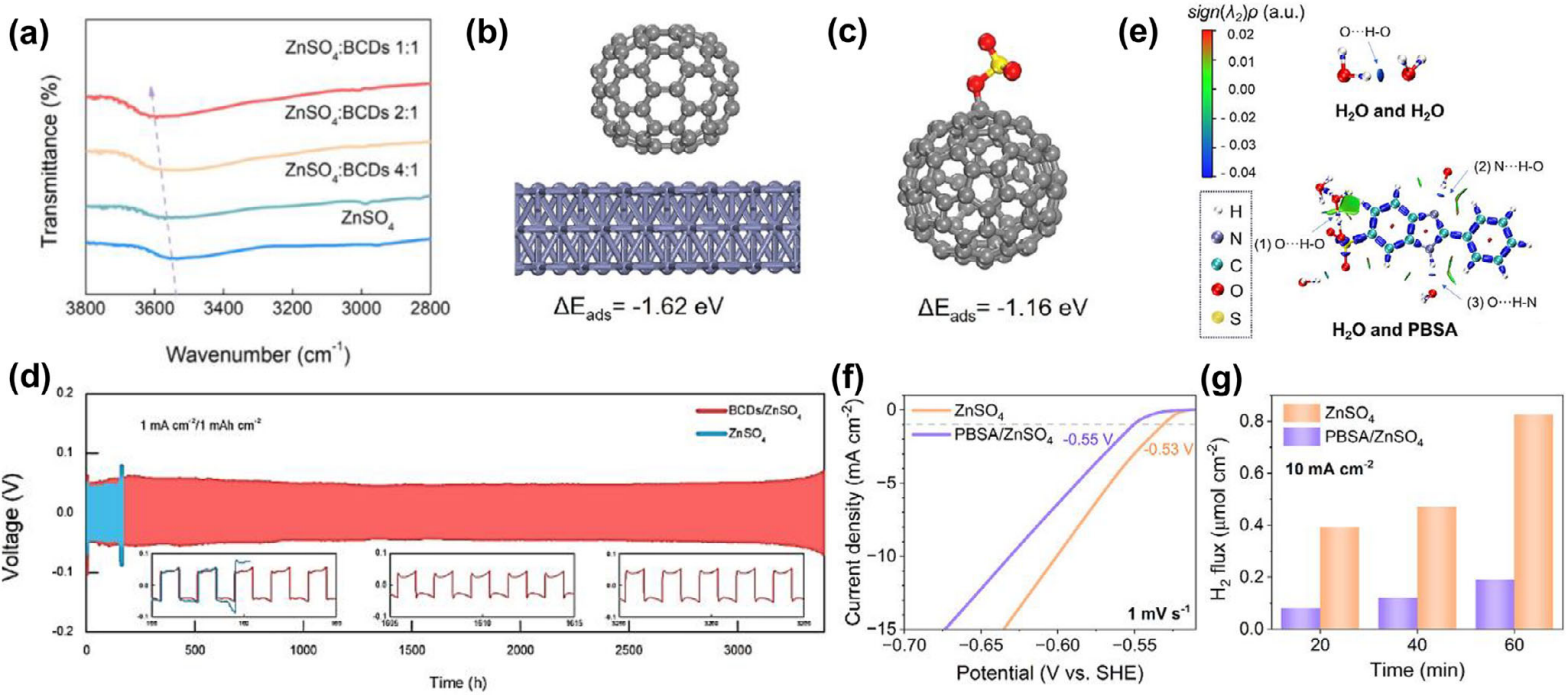
(a) FITR spectra of different electrolytes. Reprinted with permission from Ref [108]. Copyright 2025 Elsevier. The adsorption structures for (b) BCDs on Zn (002) crystal plane and (c) BCDs‐SO_4_^2−^. Reprinted with permission from Ref [108]. Copyright 2025 Elsevier. (d) Cycling performances of Zn||Zn symmetric cells with/without BCDs. Reprinted with permission from Ref [108]. Copyright 2025 Elsevier. (e) Isosurface maps of IRI of H_2_O and H_2_O and H_2_O and PBSA molecule systems. Reprinted with permission from Ref [109]. Copyright 2025 Elsevier. (f) LSV curves and (g) gas chromatography analysis of hydrogen evolution of ZnSO_4_ and PBSA/ZnSO_4_ electrolytes. Reprinted with permission from Ref [109]. Copyright 2025 Elsevier.

Liu et al. introduced green biomass carbon quantum dots (BCDs) as an electrolyte additive [108]. On the one hand, the H‐bonding network of H_2_O is strengthened. As evidenced by the FTIR spectrum in Figure 9a, with the increasing of BCD proportion, absorption peaks exhibit a red shift and diminished intensity. This indicates that BCDs form H‐bonds with H_2_O molecules via their hydroxyl groups, thereby weakening intermolecular interactions between H_2_O molecules. Consequently, this reduces H_2_O activity and suppresses the occurrence of HER side reactions. Concurrently, through a “buffered redistribution” mechanism, SO_4_^2^^−^ ions anchor onto BCDs and temporarily capture Zn^2^⁺ ions. This indirectly regulates the Zn deposition rate, delaying the deposition process and affording time for ions to migrate towards the most stable (002) crystal plane (Figure 9b,c). Ultimately, the Zn||Zn symmetric cell achieves an ultra‐long stable cycle of 3450 h (Figure 9d). Ma et al. utilized 2‐phenylbenzimidazole‐5‐sulfonic acid (PBSA) as an electrolyte additive [109]. This compound, featuring a sulfonic acid group and an azomethine ring structure, preferentially adsorbs onto Zn foil surfaces. It forms strong H‐bonds with interfacial H_2_O molecules to construct a coordination layer, disrupting continuous H‐bonding networks and impeding proton hopping. This increases the adsorption free energy of H^+^ ions, significantly inhibiting the HER. Interaction region indicator (IRI) analysis indicates that PBSA establishes multi‐point strong H‐bond interactions with H_2_O molecules (Figure 9e). Consequently, the HER potential of the PBSA/ZnSO_4_ electrolyte exhibits a pronounced negative shift (Figure 9f). Even with extended reaction times, hydrogen evolution rate and total flux remain at low levels under a constant current density of 10 mA cm^−^^2^ (Figure 9g).

**FIGURE 10:**
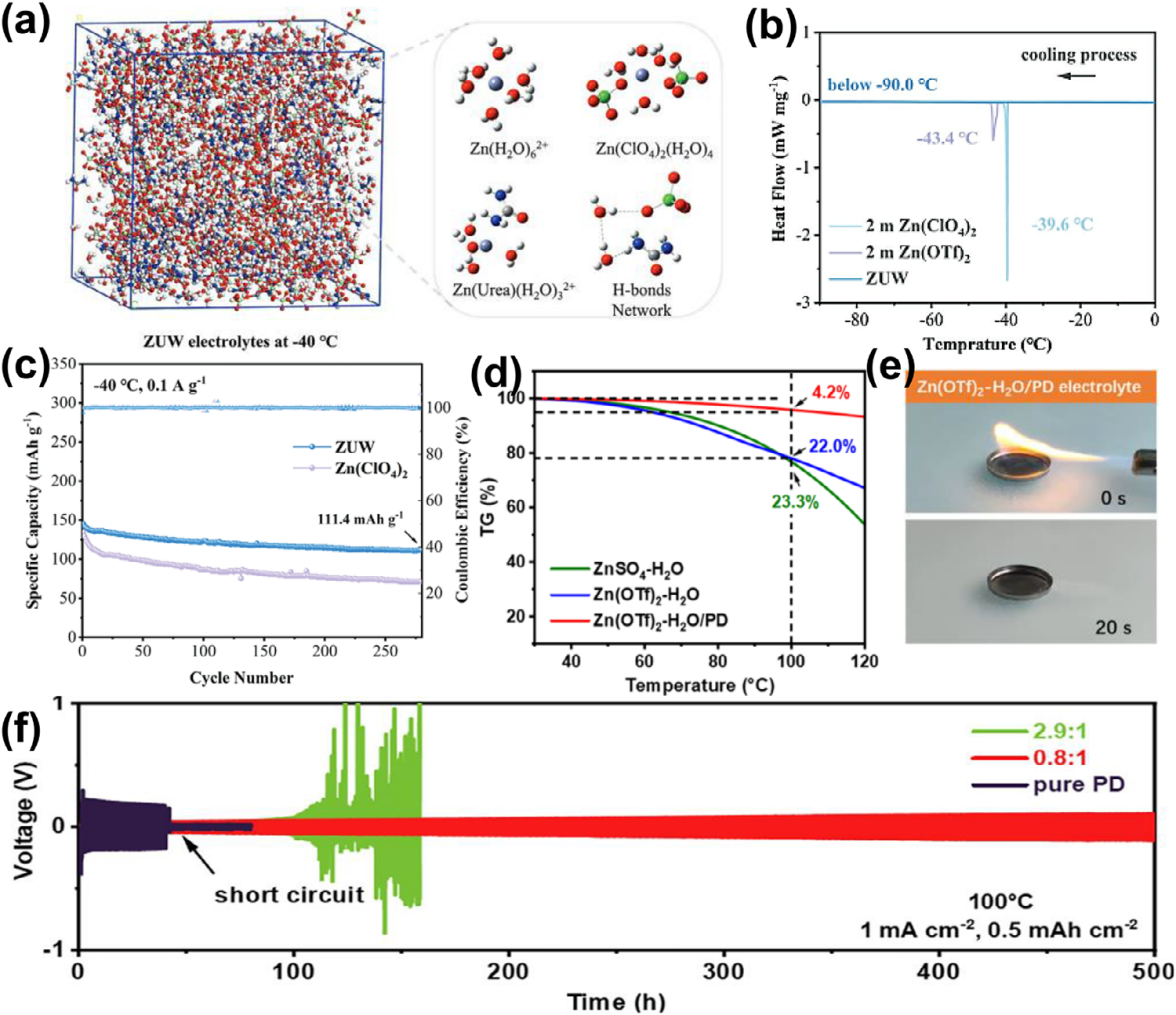
(a) The snapshots of the MD simulation and the electrolyte structure of ZUW at −40°C. Reprinted with permission from Ref [110]. Copyright 2022 Wiley‐VCH. (b) The DSC curves of different electrolytes. Reprinted with permission from Ref [110]. Copyright 2022 Wiley‐VCH. (c) Cycling performances of Zn||VO_2_ full cells at −40°C. Reprinted with permission from Ref [110]. Copyright 2022 Wiley‐VCH. (d) TG curves of different electrolytes. Reprinted with permission from Ref [111]. Copyright 2022 American Chemical Society. (e) Ignition test of Zn(OTf)_2_‐H_2_O/PD electrolyte. Reprinted with permission from Ref [111]. Copyright 2022 American Chemical Society. (f) Voltage profiles of Zn||Zn symmetric cells at 100°C. Reprinted with permission from Ref [111]. Copyright 2022 American Chemical Society.

The modulation of the H‐bonding network also contributes to the operation of the battery at wide‐temperatures. Hou et al. used Zn(ClO_4_)_2_ as a zinc salt and added urea as an additive to enhance the antifreeze performance of aqueous electrolyte through the synergistic chaotropic effect of the ClO_4_^−^ ion and urea [110]. The molecular dynamics (MD) simulation shows the synergistic chaotropic mechanism more intuitively (Figure 10a). After the addition of urea, the H‐bonds between H_2_O molecules are partially replaced by ClO_4_^−^ ions and urea, and it is difficult to find continuous H‐bonds, which effectively hinders the H_2_O molecules from arranging into a solid state at low temperature, lowering its freezing point below −90°C (Figure 10b). Moreover, the regulation of the solvation structure of Zn^2+^ ions greatly reduces the occurrence of HER. Therefore, the Zn||VO_2_ full cell has a high specific capacity of 111.4 mAh g^−1^ at −40°C (Figure 10c). Wang et al. incorporated 1,5‐pentanediol (PD) into Zn(OTf)_2_ electrolyte to construct a crowded co‐solvent [111]. By strengthening the O─H bond and excluding H_2_O from the primary Zn^2^⁺ ion solvation shell, they significantly reduce the activity of H_2_O molecules at elevated temperatures. Thermogravimetric (TG) and ignition tests demonstrate the electrolyte's non‐flammability and thermal stability (Figure 10d,e). Uniform reversible H_2_O deposition is achieved at elevated temperatures. The Zn||Zn symmetric cell exhibits stable cycling for 500 cycles at 100°C (Figure 10f).

### 4.3 Current Challenges and Future Outlooks

Electrolyte additives have gained much attention as an efficient modulation strategy to enhance electrolyte stability. However, research in this field still faces some key challenges. First, most of the additives have poor long‐term stability in a strongly polar aqueous solution environment. They are prone to oxidation, reduction, hydrolysis, or irreversible reactions with the electrode interfaces during the charging and discharging process, leading to a gradual attenuation of their protective effects and even inducing side reactions [112]. Second, it is also important to extend the electrochemical stabilization window (EWS) of the electrolyte. In addition, there is a complex synergistic and competitive relationship between the components in the electrolyte. Some additives may complex with the main salt or solvent, leading to a decrease in ion mobility, conductivity, or even cause precipitation or viscosity increase [113]. More importantly, there is still a lack of in‐depth understanding of the mechanism of additives in a dynamic electrochemical environment. Most of the studies still remain in the static analysis, while in the actual working conditions, the electrolyte interface is constantly changing with the cycle. The pH fluctuation, electric field reconstruction, and ion migration may affect the behavior of additives, which in turn affects the overall stability of the electrolyte and the reaction path [114].

To address the above challenges, future research can be carried out in the following directions. First, functional molecules with stable structure, anti‐oxidation, and anti‐hydrolysis capabilities should be developed to enhance their stability under extreme electrochemical conditions [115]. Second, additives based on H‐bonding network modulation are expected to effectively enhance the decomposition voltage of the electrolyte and broaden the ESW [116]. Third, additives synergistic modulation strategy can realize the comprehensive modulation of the electrolyte/electrode interface [117]. Fourth, with the help of DFT calculations, MD simulations, and in situ characterization (e.g., in situ FTIR, in situ XRD, etc.), the mechanism of the additive molecules can be understood in depth [118]. Finally, renewable, nontoxic, and richly sourced natural bio‐based additives can be developed from the perspective of greening and large‐scale application [119].

## 5 Summary and Future Outlooks

Electrolyte additives have garnered significant attention in recent years as key performance regulators within AZIBs systems. These additives not only effectively mitigate known performance degradation issues such as Zn dendrite growth, cathode dissolution, and side reactions, but also further modulate interfacial ion transport behavior, adjust solvation structures, and dynamically buffer electrolyte pH fluctuations, particularly under demanding electrochemical conditions like high‐rate charging/discharging or low temperatures. The diverse chemical structures of additives, ranging from small‐molecule organics and metal complexes to supramolecular frameworks, offer extensive design space for achieving multifunctional synergistic regulation. Consequently, electrolyte additive engineering serves not only as a “booster” for performance enhancement but also as a “core driver” for expanding the operational window and practical application scenarios of AZIBs. This paper systematically reviews the functional mechanisms, representative research advances, and core challenges of different additive types across three aspects: anode protection, cathode protection, and electrolyte stabilization. It aims to provide systematic theoretical support and research references for constructing high‐performance, highly stable AZIBs with extended cycle life.

In general, the application of electrolyte additives in AZIBs will gradually change from “performance improvement” to “system synergistic optimization,” not only to solve the problem of a single electrode or electrolyte component, but also to explore more versatile control strategies from the overall system stability and energy efficiency. It is also necessary to explore more versatile, intelligent, and sustainable control strategies from the perspective of overall system stability and energy efficiency.

### 5.1 Future Research Directions

Based on the current research trends and application needs, future research can continue to advance in the following directions:

1. Multi‐interface, system‐level synergy. Multifunctional additives capable of simultaneously stabilizing the Zn anode, protecting the cathode, and tuning electrolyte properties are critical. Such designs must balance multiple roles without introducing cross‐interface interference, potentially through integrating pH buffering, metal‐ion complexation, H‐bonding network modulation, and interface passivation into a single molecular framework to achieve coordinated optimization across the entire cell.
2. Development of smart responsive additives. In response to the dynamic changes in the operating environment of AZIBs (e.g., voltage fluctuations, pH drift, ion migration gradients), smart additives with self‐sensing and self‐regulation capabilities, such as electric field responsive, pH responsive, or temperature sensitive molecules, should be introduced. Such additives can automatically adjust their behaviors according to environmental changes during cell operation to achieve interface self‐healing, side reaction inhibition, and dynamic regulation of the electrochemical window.
3. Enhancing the application of in situ and operando characterization technologies. Additives often exist in very low concentrations in AZIBs systems, and their actual behavior and reaction paths are difficult to analyze by traditional means. In the future, the distribution, transformation, and interfacial behavior of additives should be monitored in real time with the help of advanced characterization methods, such as in situ Raman, in situ XPS, in situ FTIR, synchrotron radiation, etc., in order to accurately assess their regulatory effects and failure mechanisms.
4. Realizing the unity of green and scale application. Most of the current high‐efficiency additives still have problems such as high toxicity, high cost, or complex synthesis, which make it difficult to meet the needs of practical applications. Therefore, priority should be given to the development of green additives that are widely sourced, environmentally friendly and biodegradable, such as the design of additives based on natural molecules such as amino acids, peptides, polyphenols, alkaloids, etc., which will not only help to reduce the burden on the environment, but also conform to the direction of sustainable development of future energy storage technologies.
5. Apply machine learning. Screening electrolyte additives through machine learning represents a powerful strategy that elevates traditional trial‐and‐error approaches to rational design, proving crucial for guiding molecular design. Its core lies in establishing quantitative predictive models linking “additive molecular structure—microscopic interaction mechanisms—macroscopic battery performance,” thereby rapidly identifying promising targets from vast candidate molecules between synthesis and testing. This approach does not replace experimentation or mechanism studies but serves as a powerful compass, deeply integrated with computational chemistry and electrochemical characterization. However, its development remains in its infancy, necessitating efforts to promote data sharing and establish databases within the field.

### 5.2 Industrialization Challenges

Although electrolyte additives demonstrate exceptional performance enhancement potential at the laboratory scale, their commercialization still requires overcoming barriers related to economic viability and engineering‐scale amplification. First, cost‐benefit analysis serves as the primary criterion for evaluating any additive. An ideal electrolyte additive should significantly improve performance, such as extending cycle life by an order of magnitude, while having a negligible impact on the overall battery cost. Currently, many highly effective additives still face challenges such as complex synthesis routes, low yields, or expensive raw materials. Moving forward, developing additives based on biomass derivatives and industrial byproducts represents a key pathway to controlling costs and ensuring raw material availability. Second, large‐scale production requires a combination of stability and consistency. The synthesis and testing of milligram‐level batteries in laboratories cannot reflect potential issues with purity and batch stability that may arise during industrial‐scale ton‐level production. Finally, battery manufacturing must be compatible with existing battery production processes. Additives should not affect the fundamental properties of the electrolyte (such as viscosity, conductivity, etc.), nor should they impose additional environmental burdens or complex recycling processes during battery recycling. Therefore, future research on electrolyte additive performance should incorporate evaluations closer to real‐world operating conditions (e.g., low N/P ratio, high areal capacity, wide‐temperature cycling). Preliminary techno–economic analyses and environmental assessments should also be conducted to provide more robust data support for transitioning additives from the laboratory to the marketplace.

In summary, the research on electrolyte additives is at a critical stage of transition from basic exploration to engineering application, and its systematic, interdisciplinary, and forward‐looking nature will be increasingly prominent. Through continuous material innovation, mechanism research, and system integration, electrolyte additives will play a more central role in promoting high‐performance, low‐cost, green, and sustainable AZIBs technology in the future.

## Conflicts of Interest

The authors declare no conflicts of interest.

## Data Availability

The authors have nothing to report.
